# Risk factors associated with self-rated health among elderly females with different visual abilities in Chinese urban areas: a population-based study

**DOI:** 10.1186/s12889-024-19514-6

**Published:** 2024-07-23

**Authors:** Lin Su, Wei Yang, Jinsong Han, Yijiao Wu, Qiong Xie, Guowei Pan, Wei Sun, Tao Hong

**Affiliations:** 1 Department of Pain Management, Shengjing Hospital of China Medical University, Shenyang, Liaoning 110004 China; 2 Research Center for Universal Health, School of Public Health, China Medical University, No. 77 Puhe Road, Shenyang North New Area, Shenyang, 110122 PR China; 3 Department of Thoracic Surgery Medicine, Shengjing Hospital of China Medical University, Shenyang, Liaoning 110004 China

**Keywords:** Self-rated health, Visual ability, Depressive symptom, Going out alone to distant places, Elderly females, China

## Abstract

**Objective:**

Self-rated health (SRH) has been documented as an important predictor of quality of life among the elderly and its risk factors are vision-specific among elderly males. The aim of this study was to clarify vision-specific risk factors to SRH among elderly females without dementia in Chinese urban areas.

**Methods:**

From March to November 2012, 2147 elderly women in Liaoning Province of China were selected using a stratified sampling method. After cognitive screening, 1956 participants without dementia were finally enrolled. A questionnaire including SRH, visual ability and factors including demographic characteristics, physical conditions, lifestyle factors, social psychological status and social activities were analyzed. Multivariate logistic regression was used to clarify the association of SRH with risk factors, while stepwise multivariate logistic regression was used to examine the vision-specific associations with SRH.

**Results:**

The mean age was 73.6 ± 5.82 (mean ± SD). The percentages of good SRH in good and impaired visual ability groups were 36.2% and 24.4%, respectively. Most characteristics between elderly females with different visual abilities were significantly different. Visual ability had interactions with physical conditions, lifestyle factors and social activities to affect SRH. Among elderly females with good visual ability, depressive symptoms, rather than chronic disease had the strongest association with good SRH followed by marital status, regular diet, going out alone to distant places, taking a walk, smoking and alcohol consumption. In the impaired visual ability group, going out alone to distant places had the strongest association with good SRH followed by chronic disease, filial piety, taking a walk, participating in entertainment, ethnicity, quality of sleep, worrying about falling and alcohol consumption.

**Conclusions:**

Good SRH status was at a low level especially among elderly females with impaired visual ability and the risk factors differed between elderly females with different visual abilities. Social psychological status was crucial for SRH among elderly females with good visual ability whereas physical conditions were prominent for impaired visual ability group.

## Background

Population aging, which has become a global issue in the 21st century [1], brings great burden to society with its rapid growth. As reported by the National Bureau of Statistics of China, from 2000 to 2020, the percentage of people aged 65 years and over increased from 7.0 to 13.5%, but the development of the economic and health care systems have not kept pace with the aging of the population. As the elderly consume a major proportion of medical resources and services [2, 3], it has become urgent to maintain and improve the quality of life of the elderly.

Self-rated health (SRH) is proposed as an authentic predictor of disability and mortality of the elderly which integrates the physical, psychological and social functions of an individual [4]. A three-year prospective cohort study had proved the SRH to be an independent predictor of functional decline in community-dwelling elderly [5]. In-depth studies of SRH and its risk factors had been well conducted among the elderly worldwide [6–12]. Chronic disease, depressive symptoms, physical exercise, functional status, obesity, mobility difficulties, diet, family relationships and socioeconomic factor have been revealed to be the risk factors to SRH in previous studies [13].

Visual ability has been proved to be a major health issue for the elderly [14]. As a part of physical functioning, it affects quality of life directly and indirectly through worsening mobility and psychological health [15–18]. Considering its considerable effect on the independent living ability of the elderly [18], it is important to study the effects of SRH and its risk factors on elderly individuals with different visual abilities to provide prompt and proper health care. In our previous study conducted among elderly males [19], we proved that physical condition was prominent for SRH among the elderly males with good visual ability, whereas lifestyle factors were crucial for those with impaired visual ability. As for elderly females, we also hypothesized that the risk factors for SRH tended to be vision-specific. Since females have a longer life expectancy by 5.79 years than men in China [20], it would seem to be important to perform the assessment among elderly females. In previous studies, cognitive function screening among the elderly population was not examined sufficiently. The elderly with diagnosed dementia recognized. However, the elderly who suffer from cognitive impairment, but not as serious as dementia, might be included in study populations. This fact would cause the information bias and inevitably weaken the application of conclusions. Thus, performing assessment on cognitive impairment emerged to be an important issue while conducting survey among the elderly.

The current study was designed to evaluate SRH and clarify visual-specific risk factors among elderly females without cognitive impairment. It initially focused on elderly females in urban areas of China. The mini-mental state examination (MMSE) was explored to assess cognitive function. SRH, visual ability, factors including demographic characteristics, physical condition, lifestyle factors, social psychological status and social activities were measured after cognitive function screening, with the outcomes used to benefit health improvements for the elderly.

## Materials and methods

### Study area and study population

The present study was conducted in Shenyang city and Anshan city in Liaoning province in China where the average income level was similar to the national average [15]. A stratified cluster random sampling method was adopted, with four districts randomly selected from two cities and one community randomly selected in each sampling district. Detailed study designs can be found in previously published articles [19]. All 2480 females who were at least 65 years of age, had no diagnosed dementia, lived in the local area over five years and consented to complete this study were interviewed. There were 2147 effective responses obtained (effective response rate, 86.6%) while 1956 participants became our subjects after cognitive function screening, which was based on the MMSE. Data collection occurred during March to November 2012.

### Cognitive function screening

In this study, MMSE was used to screen cognitive functions in elderly females. The total score ranged from zero to 30 and the MMSE included 19 items that fully reflected the abilities of orientation, registration, calculation, recall, naming, repetition, comprehension, writing and construction. Based on the previous MMSE assessment results of the Chinese elderly population, the MMSE score thresholds for cognitive impairment were set as follows: ≤17, illiterate; ≤20, elementary; ≤22, middle/high school and ≤ 23 for junior college and over [19, 21, 22].

### Assessment of SRH

The assessment of SRH, which has been applied in our prior study among elderly males in China and non-disabled elderly in Japan, was applied to this study [12, 19]. It was evaluated by asking: ‘what do you think of your health in general?’ with the responses dichotomized as ‘good’ or ‘poor’.

### Measurements of visual ability and associated factors including demographic characteristics, physical condition, lifestyle factors, social psychological status and social activities

Visual ability was assessed based on subjective rather than objective measures, such as the measurement of visual acuity, field and sensitivity. Previous studies have shown that subjective measurements can better reflect global vision problems in the elderly, such as difficulty seeing and reading and had an important influence on the daily life of an individual [23]. Newspapers were prepared and participants were asked to read out certain paragraphs with or without glasses according to their daily life habits. As for the illiterate elderly, they were asked to read the number included in the newspaper and respond the clearness of watching. Those who could read the words and numbers in the newspaper correctly were defined as ‘good’ and those who could not read correctly were defined as ‘poor’ [19].

The demographic characteristics included the following six items: (1) age; (2) ethnicity; (3) marital status; (4) living arrangement; (5) pension; and (6) health insurance. The responses of marital status including ‘single’, ‘divorced’, ‘separated’ or ‘widowed’ were merged as ‘other’ group due to their low ratios, less than 1.0%.

The physical condition was evaluated based on the following five items: (1) chronic disease; (2) hearing ability; (3) quality of sleep; (4) going out alone to distant places; and (5) worrying about falling. The above five items were measured by referring to our previous study conducted in Japan and China [12, 19].

The lifestyle factors were assessed by the following four items: (1) taking a walk; (2) smoking; (3) alcohol consumption; and (4) regular diets. Taking a walk was measured by the frequency which was classified as ‘≤1–2 times/week’ or ‘≥3–4 times/week’. Smoking and alcohol consumption were defined as ‘yes/had ever’ or ‘never/no’. Regular diets were assessed whether breakfast, lunch and dinner were eaten on time [12, 19].

The social psychological status was comprised of the following two items: (1) depressive symptoms and (2) filial piety. Depressive symptoms were assessed by the Geriatric Depression Scale (GDS-15) [24, 25]. Filial piety was evaluated by asking: ‘What do you think about your children’s filial piety to you?’ with the responses being dichotomized as ‘good’ (very good/good) and ‘poor’ (so so/bad) [19].

The social activities were assessed based on the following two items: (1) participating in entertainment and (2) watching television frequently. Participating in entertainment assessed the state of an individual frequently playing mahjong, chess, or cards with others, or looking around. ‘Do you often watch television frequently?’ was designed to assess the item of watching television frequently, with the answer defined as ‘yes’ or ‘no’ [19].

### Statistical analysis

Date analysis was operated respectively in different visual ability groups. Given that over 96.9% of the participants had the same responses, health insurance in both groups was excluded in the data analysis.

The chi-square test served to test the differences in subject characteristics between different visual ability groups and the distribution of SRH among the categorical variables. Multivariate logistic regression was used to clarify the association of SRH with risk factors. Stepwise multivariate logistic regression was used to examine the vision-specific associations with SRH. Risk factors to SRH were further explored among elderly females with good visual ability and impaired visual ability based on the prominent difference of characteristics in different visual ability groups.

The data are presented as number (N), prevalence (%), OR and 95% CI. Age was treated as a continuous variable and was shown as mean ± SD. In this study, the missing data rate was less than 5%, and there was no need to handle missing data. SPSS 13.0 was used to perform data analysis.

## Results

The average age of 1956 elderly females was 73.6 ± 5.82 (mean ± SD). The average age of good (72.5 ± 5.62) and the impaired (75.0 ± 5.75) visual ability groups was significantly different (*p* < 0.05). The comparison of subject characteristics between good and impaired visual ability groups is shown in Table 1. The prevalence of good SRH in the good visual ability group (36.2%) was higher than that in the impaired visual ability group (24.4%) (*p* < 0.01) and the differences between them were statistically significant among most items of demographic characteristics, physical conditions, lifestyle factors, social psychological status, as well as social activities (*p* < 0.05).

**Table 1:** The comparison of subject characteristics between good and impaired visual ability groups

Variables	Good visual ability *N* (%)	Impaired visual ability *N* (%)	χ^2^	*p*
**Good SRH**	402(36.2)	206(24.4)	30.89	0.000
**Demographic characteristics**				
Age				
75+	352(31.7)	460(54.5)		
65–74	760(68.3)	384(45.5)	103.16	0.000
Ethnicity				
Minority	97(8.7)	172(20.4)		
Han	1014(91.3)	672(79.6)	54.84	0.000
Marital status				
Other	211(19.0)	259(30.7)		
Married/cohabitation	901(81.0)	585(69.3)	36.06	0.000
Living arrangement				
Living alone	134(12.1)	92(10.9)		
Living with others	976(87.9)	750(89.1)	0.61	0.433
Pension				
Haven’t	67(6.0)	121(14.3)		
Have	1042(94.0)	723(85.7)	37.91	0.000
**Physical conditions**				
Chronic disease				
Present	801(72.0)	724(85.8)		
Not present	311(28.0)	120(14.2)	52.80	0.000
Hearing ability				
Impaired	288(25.9)	565(67.0)		
Good	824(74.1)	278(33.0)	329.70	0.000
Quality of sleep				
Impaired	488(43.9)	479(56.8)		
Good	624(56.1)	365(43.2)	31.79	0.000
Going out alone to distant places				
Cannot	611(55.0)	681(80.8)		
Can	501(45.1)	162(19.2)	142.82	0.000
Worrying about falling				
Yes	452(40.7)	417(49.5)		
No	658(59.3)	425(50.5)	15.03	0.000
**Lifestyle factors**				
Taking a walk				
≤ 1–2 times/week	367(33.0)	336(39.8)		
≥ 3–4 times/week	745(67.0)	508(60.2)	9.66	0.002
Smoking				
Yes/had ever	279(25.1)	168(19.9)		
Never	833(74.9)	675(80.1)	7.24	0.007
Alcohol consumption				
Yes	162(14.6)	80(9.1)		
No	950(85.4)	762(90.5)	11.34	0.001
Regular diet				
No	151(13.6)	138(16.4)		
Yes	961(86.4)	706 (83.7)	2.93	0.087
**Social psychological status**				
Depressive symptom				
Present	164(14.8)	295(30.0)		
Not present	948(85.3)	591(70.0)	66.33	0.000
Filial piety				
Poor	90(8.1)	75(9.0)		
Good	1018(91.9)	759(91.0)	0.46	0.496
**Social activities**				
Participating in entertainment				
No	357(32.2)	457(54.3)		
Yes	752(67.8)	384(45.7)	96.49	0.000
Watching television frequently				
No	154(13.9)	129(15.3)		
Yes	956(86.1)	714(84.7)	0.79	0.374

Univariate analysis of factors in relation to good SRH among elderly females with different visual ability groups is shown in Table 2. For participants with good visual ability, factors including all terms of physical conditions except hearing ability, all terms of lifestyle factors except smoking, all terms of social psychological status and participating in entertainment, were significantly related to good SRH (*p* < 0.05). In contrast, the good SRH of elderly females with impaired visual ability, was correlated with ethnicity, marital status and all items of physical conditions, lifestyle factors, social psychological status, as well as social activities (*p* < 0.05). The OR value of depressive symptoms (OR = 6.35) was the highest in the good visual ability group, while going out alone to distant places (OR = 12.38) was the strongest in impaired visual ability group.

**Table 2:** Univariate analysis of factors related to good SRH among elderly females with different visual ability

Variables	Good visual ability (*N* = 1112)		Impaired visual ability (*N* = 844)	
	Good SRH *N* (%)	OR (95%CI)	Good-SRH *N* (%)	OR (95% CI)
**Demographic characteristics**				
Age				
75+	99(28.1)		62(13.5)	
65–74	303(39.9)	1.69(1.29–2.23)	144(37.5)	3.85(2.75–5.40)
Ethnicity				
Minority	40(41.2)		24(14.0)	
Han	362(35.7)		182(27.1)	2.29(1.44–3.64)
Marital status				
Other	49(23.2)		29(11.2)	
Married/cohabitation	353(39.2)	2.13(1.51–3.01)	177(30.26)	3.44(2.25–5.26)
Living arrangement				
Living alone	31(23.1)		11(12.0)	
Living with others	371(38.0)	2.04(1.34–3.11)	195(26.0)	2.59(1.35–4.96)
Pension				
Haven’t	19(28.4)		26(21.5)	
Have	382(36.7)		180(24.9)	
**Physical conditions**				
Chronic disease				
Present	208(26.0)		137(18.9)	
Not present	194(62.4)	4.73(3.58–6.25)	69 (57.5)	5.80(3.86–8.71)
Hearing ability				
Impaired	100(34.7)		83(14.7)	
Good	302(36.7)		123(44.2)	4.60(3.31–6.42)
Quality of sleep				
Impaired	147(30.1)		66(13.8)	
Good	255(40.9)	1.60(1.25–2.06)	140(38.4)	3.89(2.79–5.44)
Going out alone to distant places				
Cannot	152(24.9)		97(14.2)	
Can	250(49.9)	3.01(2.33–3.88)	109(67.3)	12.38(8.36–18.33)
Worrying about falling				
Yes	139(30.8)		57(13.7)	
No	263(40.0)	1.50(1.16–1.93)	148(34.8)	3.37(2.39–4.76)
**Lifestyle factors**				
Taking a walk				
≤ 1–2 times/week	107(29.2)		28(8.3)	
≥ 3–4 times/week	295(39.6)	1.59(1.22–2.08)	178(35.0)	5.93 (3.87–9.10)
Smoking				
Yes/had ever	112(40.1)		28(16.7)	
Never	290(34.8)		178(26.4)	1.79(1.15–2.78)
Alcohol consumption				
Yes	71(43.8)		29(36.3)	
No	331(34.8)	0.69(0.49–0.96)	176(23.1)	0.53(0.32–0.86)
Regular diet				
No	36(23.8)		16(11.6)	
Yes	366(38.1)	1.97(1.32–2.92)	190(26.9)	2.81(1.62–4.85)
**Social psychological status**				
Depressive symptom				
Present	16(9.8)		85(33.6)	
Not present	386(40.7)	6.35(3.73–10.81)	121(20.5)	0.51(0.37–0.71)
Filial piety				
Poor	20(22.2)		6(8.0)	
Good	380(37.3)	2.08(1.25–3.48)	199(26.2)	4.09(1.75–9.56)
**Social activities**				
Participating in entertainment				
No	98(27.5)		54(11.8)	
Yes	304(40.4)	1.79(1.36–2.36)	150(39.1)	4.78(3.37–6.79)
Watching television frequently				
No	57(37.0)		21(16.3)	
Yes	345(36.1)		185(25.9)	1.80(1.09–2.96)

The OR value was shown when the difference was significant

The multivariate logistic regression analysis of the factors associated with good SRH among the total population is shown in Table 3. With adjustment for age, the factors significantly associated with good SRH were chronic disease (not present), living arrangement (living with others), going out alone to distant places (can), taking a walk (≥ 3–4 times/week), participating in entertainment (yes), regular diet (yes), quality of sleep (good), and alcohol consumption (yes) in the sequence of OR value. However, the association between SRH and alcohol consumption was reversed and was not significant between SRH and visual ability.

**Table 3:** The multivariate logistic regression analysis of factors associated with good SRH among the total population

Variables	Beta	Walds	OR (95%CI)	*p*
Age (years) ^a^	-0.05	26.74	0.95 (0.93–0.97)	< 0.01
Chronic disease (not present vs. present)	1.20	76.85	3.31 (2.53–4.33)	< 0.01
Living arrangement (living with others vs. living alone)	0.85	17.15	2.34 (1.57–3.50)	< 0.01
Going out alone to distant places (can vs. cannot)	0.73	31.35	2.08 (1.61–2.68)	< 0.01
Taking a walk (≥ 3–4 times/week vs. ≤ 1–2 times/week)	0.58	19.53	1.79 (1.38–2.32)	< 0.01
Participating in entertainment (yes vs. no)	0.55	19.90	1.73 (1.36–2.20)	< 0.01
Regular diet (yes vs. no)	0.46	6.42	1.58 (1.11–2.25)	< 0.01
Quality of sleep (good vs. impaired)	0.27	5.04	1.31 (1.03–1.65)	0.02
Alcohol consumption (no vs. yes)	-0.73	19.00	0.48 (0.35–0.67)	< 0.01

^a^ age was treated as continue variable and fixed in the model

Stepwise multivariate logistic regression analysis of vision-specific associations with good SRH is shown in Table 4. The items that were significant among the total population and their interactions with visual ability were entered into the model. The interactions between visual ability and physical conditions (going out alone to distant places), lifestyle factors (alcohol consumption, regular diet, and taking a walk), and social activities (participating in entertainment) were significant (*p* < 0.05).

**Table 4:** Stepwise multivariate logistic regression analysis of vision-specific associations with good SRH

Variables	OR	
	Value	95%CI
Total population (*N* = 1943)		
Age (years) ^a^	0.95	0.93–0.97
Going out alone to distant places (can vs. cannot)	5.56	3.53–8.76
Chronic disease (not present vs. present)	3.68	2.79–4.84
Taking a walk (≥ 3–4 times/week vs. ≤ 1–2 times/week)	3.06	1.89–4.95
Living arrangement (living with others vs. living alone)	2.34	1.57–3.50
Participating in entertainment (yes vs. no)	2.23	1.47–3.38
Visual ability (good/impaired)* Alcohol consumption (no vs. yes)	1.64	1.18–2.28
Visual ability (good/impaired)* Regular diet (yes vs. no)	1.60	1.05–2.46
Visual ability (good/impaired)* Participating in entertainment (yes vs. no)	0.57	0.35–0.95
Visual ability (good/impaired)* Taking a walk (≥ 3–4 times/week vs. ≤ 1–2 times/week)	0.44	0.25–0.78
Alcohol consumption (no vs. yes)	0.37	0.25–0.54
Visual ability (good/impaired)* Going out alone to distant places (can vs. cannot)	0.28	0.16–0.47

^a^ age was treated as continue variable and fixed in the model

The results of the multivariate logistic regression analysis of the factors related to good SRH among the elderly females with different visual ability are shown in Table 5. Age was adjusted in the model and the associations were tabled in the OR sequence. Factors including depressive symptoms (present), chronic disease (not present), marital status (married), regular diet (yes), going out alone to distant places (can), taking a walk (≥ 3–4 times/week), smoking (had ever), and alcohol consumption (yes) were found to associate with good SRH in the good visual ability group. Going out alone to distant places (can), chronic disease (not present), filial piety (good), taking a walk (≥ 3–4 times/week), participating in entertainment (yes), ethnicity (Han), quality of sleep (good), worrying about falling (no), and alcohol consumption (yes) were significant for the impaired visual ability group.

**Table 5:** The multivariate logistic regression analysis of factors associated with good SRH with different visual ability

Variables	Beta	Walds	OR (95%CI)	*p*
**Good visual ability** (*N* = 1112)				
Age (fixed) ^a^	-0.03	6.47	0.97 (0.94–0.99)	0.01
Depressive symptom (present vs. not present)	1.29	20.38	3.64 (2.08–6.37)	< 0.01
Chronic disease (not present vs. present)	1.21	58.64	3.35 (2.46–4.56)	< 0.01
Marital status (married/cohabitation vs. other)	0.54	7.25	1.72 (1.16–2.54)	< 0.01
Regular diet (yes vs. no)	0.47	4.31	1.60 (1.03–2.51)	0.04
Going out alone to distant places (can vs. cannot)	0.39	5.85	1.48 (1.08–2.03)	0.02
Taking a walk (≥ 3–4 times/week vs. ≤ 1–2 times/week)	0.34	4.35	1.41 (1.02–1.94)	0.04
Smoking (never vs. yes/had ever)	-0.46	7.14	0.63 (0.45–0.88)	< 0.01
Alcohol consumption (no vs. yes)	-0.49	5.40	0.61 (0.41–0.93)	0.02
**Impaired visual ability** (*N* = 827)				
Age (fixed) ^a^	-0.05	7.13	0.95 (0.91–0.99)	< 0.01
Going out alone to distant places (can vs. cannot)	1.45	33.32	4.28 (2.61–7.01)	< 0.01
Chronic disease (not present vs. present)	1.16	14.71	3.19 (1.76–5.76)	< 0.01
Filial piety (good vs. poor)	1.13	4.74	3.09 (1.12–8.52)	0.03
Taking a walk (≥ 3–4 times/week vs. ≤ 1–2 times/week)	1.00	15.15	2.72 (1.64–4.50)	< 0.01
Participating in entertainment (yes vs. no)	0.97	17.91	2.64 (1.68–4.13)	< 0.01
Ethnicity (Han vs. minority)	0.88	8.31	2.42 (1.33–4.41)	< 0.01
Quality of sleep (good vs. impaired)	0.74	11.30	2.09 (1.36–3.21)	< 0.01
Worrying about falling (no vs. yes)	0.49	4.44	1.63 (1.04–2.56)	0.04
Alcohol consumption (no vs. yes)	-1.05	10.09	0.35 (0.19–0.67)	< 0.01

^a^ age was treated as continue variable

## Discussion

The study population comprised 1956 community-dwelling elderly Chinese females. In comparison to our previous study on elderly males [19], the sample size was also more than a quarter out of the total female inhabitants in sampled areas, ensuring good representation of the study population. In this study, differences in the characteristics between female participants with different visual abilities was considerable in almost all items measured. Further vision-specific analysis revealed that the levels of good SRH differed between elderly females with good and impaired visual ability, which was in agreement with the conclusions drawn from a 4-year follow-up study [26] and the Chinese Health and Retirement Longitudinal Study [27]. Those studies also revealed that the older adults with self-reported poor vision tended to report poor SRH. Furthermore, new interactions between visual ability and other risk factors were discovered. This finding together with the reports that visual ability could affect the mobility and psychological health of the elderly [15–18] prompted us to clarify vision-specific risk factors. All these facts confirmed the hypothesis that, like elderly males, visual-specific health care might be more beneficial for the self-assessment of health status among the elderly females.

Considering the level of good SRH, the study results showed that the prevalence among elderly females with good visual ability (36.2%) was higher in comparison to those with visual impairment (24.2%). However, compared to elderly females in Shanghai (42.2%) [7], Chile (59.2%) and Brazil (55.2%) [28], the level in the good visual ability group was significantly lower. These results showed that the good SRH status of the Chinese elderly females in urban areas was still at a lower level. Due to SRH being a reliable and predictable indicator, establishing an appropriate approach for good SRH is needed to improve the quality of life of the elderly population.

Examination of risk factors showed that the capacity of going out alone to distant places was found to be the strongest risk factor associated with SRH among the elderly females with impaired visual ability, ranked fifth in the good visual ability group. This ability seems to represent the ability to live independently and perform daily and social activities individually [29]. It had been originally used as an indicator of mobility of the elderly in Japan. An elderly person who cannot go out alone to distant places tends to become housebound, has no confidence toward life, and seldom participates in social activities [30]. The ability to go out alone to distant places had been revealed to have an effect on SRH among the elderly living alone [12]. Individuals with impaired visual ability usually have fundamental living difficulties, which made them pay more attention to physical conditions. Maintaining the capacity to go out alone to distant places should therefore be prioritized for the elderly with visual impairment.

For elderly females with good visual ability, depressive symptoms turned out to be the strongest risk factor affecting good SRH. This psychological status had been proved to be associated with SRH in many existing studies [7, 28], but its effect was not found in the impaired visual ability group. This might be because people with good visual ability had less fundamental living difficulties and could participate in social activities. The effect of physical abilities tended to be weaker in comparison to psychological status when performing the subjective assessment on SRH. However, since individuals with impaired visual ability are vulnerable both physically and psychologically, improving the condition of depressive symptoms in both good and impaired visual ability groups seems to improve the level of SRH.

Chronic disease was clarified as the second important risk factor for elderly females in both good and impaired visual ability groups. The elderly were shown to be the highest risk population for chronic disease [31]. The existence of chronic disease represents poor physical condition [15] and influenced the self-rated assessment on health status, as was reported in previous studies [12, 19, 32]. In this study, even if the percentage of individuals without chronic disease in the good visual group (28%) was twice that in impaired visual group (14%), it was found that the association between chronic disease and SRH was strong in both groups and shared similar OR value at 3.35 and 3.19, respectively. Similar conclusions were also drawn in studies in Japan [12], Brazil and Chile [28], and confirmed that chronic disease could be a significant risk factor for the SRH of the participants, regardless of their visual ability. Decreasing the prevalence of chronic diseases of the elderly should be a focus for the health care system.

In China, traditional Confucian Teachings have been regarded as Chinese major spirit for thousands of years. The filial piety duty of descendants is a representation of the Chinese tradition of Confucian culture and would influence the satisfaction of life and even the perception of health status [33, 34]. Our results also revealed a strong association with good SRH among elderly females with impaired visual ability, but not among the group with good visual ability. In comparison to the elderly females with good visual ability, those with impaired visual ability tended to be frailer and need more help from their offspring, confirming higher filial expectations as a prominent influence on good SRH. In contrast, marital status was more important for those with good visual ability, with the company of her spouse more important than the filial expectation from children. The filial piety duty of children has considerable effect of on both physical and mental health of Chinese elderly [14] and is important to those with visual impairment from the viewpoint of good SRH.

Taking a walk has been recognized as a particularly feasible form of physical exercise for the aged population. From the viewpoint of perceived health status, it showed a strong association with good SRH, with an OR value of 2.72 in the impaired visual ability group and 1.41 in the good visual ability group. Compared to individuals in the good visual ability, people with impaired visual ability are more likely to report mobility difficulties [35], which was manifested as having a lower walking speed and more falls, limiting an individual’s daily activities and becoming an obstacle to engaging in physical activity. Taking a walk as a typical physical activity will affect the evaluation of health status, especially for those with impaired visual ability. Since it can contribute to the successful aging of both males and females [13], the encouragement to take a walk should be strongly given to older adults with impaired visual ability to maintain good SRH.

In this study, participating in entertainment was found to be associated with SRH among individuals with visual impairment. Social activity has been reported to be an important risk factor related to SRH among Japanese elderly individuals living alone [12] and the same conclusion was also drawn among the elderly males with different visual abilities [19]. As for the elderly females, its effect was only observed among those with visual impairment. Compared to those with good visual ability (32.2%), high absence of participation in entertainment among individuals with impaired visual ability (54.3%) seemed to be the main cause. Increasing the involvement of the social activity of the elderly with impaired visual ability should be a focus of the health promotion for the elderly.

Ethnicity was also associated with SRH and Han nationality reported higher levels of good SRH among the elderly females with visual impairment, coinciding with the conclusions drawn from other Chinese elderly [36, 37]. This may be related to the difference between Han and minorities in social support [36].

In addition to chronic disease, physical conditions such as quality of sleep and worrying about falling were shown to have effect on the SRH for elderly females with impaired visual ability, but not for those with good visual ability. Poor quality of sleep has proved to worsen an individual’s perception of SRH and independent living ability [38]. People who has visual impairment tend to have a higher possibility of falling, which seemed to aggravate their fear to go outside and then influence their perception of their health status [24].

Besides, being components of lifestyle factors, regular diet, smoking, and alcohol consumption were identified as affecting SRH. A regular diet ensures good nutrition and could contribute to good SRH [37]. Both smoking and alcohol consumption showed a significantly positive association with good SRH. This may be due to the fact that heavy smoking and drinking were barely for the elderly. On the contrary, relatively small amount of smoking and alcohol needed to satisfy the needs of the elderly [39]. Further study is needed to verify this finding.

## Limitations

There were several limitations for the present study. First, as a cross-sectional design, the causality relations between associated factors and good SRH could not be drawn, so all findings need to be proved by further prospective study. Second, the measurements of smoking and alcohol consumption were lacking in precision, which might weaken the evaluation of their effects.

## Conclusions

This population-based study was the first to assess the effects of visual-specific risk factors on SRH among the Chinese elderly females with double cognitive screenings in urban areas of China. The results showed that good SRH status was at a low level especially among elderly females with impaired visual ability and the risk factors differed between elderly females with different visual abilities. Depressive symptoms had a crucial effect on the SRH of elderly females with good visual ability; whereas going out alone to distant places was prominent for elderly females with impaired visual ability. These findings suggest that visual-specific risk factors should be a focus of health promotion in order to maintain and improve the good SRH of the elderly.

## Data Availability

The datasets used and/or analyzed during the current study are available from the corresponding author on reasonable request.

## Abbreviations

SRH: Self-Rated Health
MMSE: The Mini-Mental State Examination
